# Review of herbal medicine works in the avian species

**DOI:** 10.14202/vetworld.2021.2889-2906

**Published:** 2021-11-11

**Authors:** Tyagita Hartady, Mas Rizky A. A. Syamsunarno, Bambang Pontjo Priosoeryanto, S. Jasni, Roostita L. Balia

**Affiliations:** 1 Study Program of Veterinary Medicine, Faculty of Medicine, Universitas Padjadjaran, Jatinangor, Indonesia; 2 Department of Biomedical Sciences, Faculty of Medicine, Universitas Padjadjaran, Jatinangor, Indonesia; 3 Department of Clinic Reproduction Pathology, Faculty of Veterinary Medicine, Bogor Agricultural University, Bogor, Indonesia; 4 Department of Paraclinical, Faculty of Veterinary Medicine, Universiti Malaysia Kelantan, Kelantan, Malaysia; 5 Department of Public Health, Faculty of Medicine, Universitas Padjadjaran, Jatinangor, Indonesia

**Keywords:** antimicrobial resistance, avian herbal therapy, avian medicinal plants, chicken disease, herbal medicine, poultry herbal medicine

## Abstract

Poultry meat consumption is increasing worldwide but the overuse of antimicrobials for prevention and treatment of diseases has increased antimicrobial resistance (AMR), triggering a major public health issue. To restrict AMR emergence, the government supports the optimization of natural products that are safe and easy to obtain with minimal side effects on poultry, humans, and the environment. Various studies have explored the potential of herbs in animal health for their antiviral, antibacterial, antifungal, antiparasitic, immunomodulatory, antioxidant, and body weight gain properties. Therefore, this study reviewed plants with potential application in avian species by summarizing and discussing the mechanisms and prophylactic/therapeutic potential of these compounds and their plant origin extracts.

## Introduction

Poultry meat is consumed worldwide, but the overuse of antimicrobials to prevent and treat diseases in poultry has facilitated the emergence of antimicrobial resistance (AMR) [1]. AMR in poultry is reportedly [2] due to excessive use of antibiotics, including disease prevention programs, treatment of infections, accelerating growth, and increasing production [3]. Antibacterials are equally important drugs for treating human diseases [4]; therefore, AMR emergence in poultry requires discovering safe synthetic antibacterial substitutes.

Developing countries commonly use herbal medicines for treating various diseases [5] and herbs have become popular alternatives for poultry, as natural antibacterial and antifungal [6], antiprotozoal [7,8], antiviral [9], antioxidants, growth promoters, anti-inflammatory, and for increasing immunity and weight [10]. Herbs are potential antimicrobials due to their phenolic components (simple phenols, phenolic acids, quinones, flavones, tannins, and coumarins), terpenoids and essential oils, alkaloids and lectins, and polypeptides [11]. They are also relatively inexpensive with no harmful side effects due to simple absorption by the body. Hence, traditional remedies are widely used based on low cost, local availability, and practical application and do not require modern technology, such as refrigeration [12].

Herbs have other benefits in escalating host response to vaccination by inducing infection prevention through controllable procedures [13]. Therefore, the objective of this review is to evaluate *in vivo* studies on poultry as recommended references for scientists, field practitioners, and pharmaceutical manufacturers for poultry disease prevention and control.

## Literature Search

We used an inductive approach to search electronic research databases and identify topics related to herbal medicine compounds or extracts that affect avian species as antiviral, antibacterial, antifungal, antiparasitic, immunomodulator, antioxidant, and body weight gain agents. We retrieved and reviewed 76 relevant articles investigating components that contribute to herbal medicine action in avian species.

## Antiviral

Viruses are disease-causing agents that easily emerge, mutate, and spread, making them difficult to treat. In the poultry industry, there are several prevalent viral diseases, such as infectious bursal disease, Newcastle disease (ND), and infectious laryngotracheitis. Various vaccines are available to protect from such diseases, but most farmers prefer using herbs, as some believe that vaccination is ineffective. Further, vaccines are expensive, often have no guarantee of bird safety, and are not easily accessible and occasionally noxious [14].

Yasmin *et al*. [15] reported that herbs, such as aloe species, *Azadirachta indica* (neem) and *Commiphora swynnertonii* (Burtt), can treat ND. However, aloe species are only effective for disease management rather than treatment, as there were no significantly different effects between treated and untreated groups. Surprisingly, neem leaf extract demonstrated anti- ND virus (NDV) properties by reducing NDV-stimulated splenocyte proliferation in mice compared with uninfected controls, while administering Burtt extract resulted in significantly lower mortality rates based on clinical symptoms and antibody titer.

Ou *et al*. [16] revealed that combined extracts of *Rhizoma Dryopteridis crassirhizomatis* and *Fructus mume* are effective against infectious bursal disease virus (IBDV) infection. The extract combination improved antibody levels and decreased viral load in the targeted organs. Similarly, *Camellia sinensis* (green tea) is effective for treating avian influenza (AIV) and inclusion body hepatitis-hydropericardium syndrome. Catechins from *C. sinensis* can suppress viral RNA synthesis by inhibiting endonuclease activity of RNA polymerase, thus potentially killing the virus. The interaction between catechins and viral neuraminidase proteins can prevent virus release [17,18].

Oil extract from *Nigella sativa* oil is suitable as adjuvants for AIV vaccines, as reviewed by Mady *et al*. [19]. The herbs also improved host response to vaccination. Administration of *N. sativa* oil on 14 days post-avian influenza vaccination stimulated lymphocyte proliferation and immunity development to 86% phagocytic percent [20]. Further, virgin coconut oil promotes T-lymphocyte production, improving T-helper cells, as well as stimulating antibody production from B-lymphocyte cells against AIV. However, a similar study shows that chicks given a 15 mL VCO/kg diet can diminish lymphocytes [21].

Tannin also has antiviral properties [22]. Latheef *et al*. [23] investigated the immunomodulatory activity of *Withania somnifera*, *Tinospora cordifolia*, and *A. indica* in chicks infected with chicken infectious anemia virus, observing that the herbs effectively resisted virus multiplication and pathogenesis. A combination of extracts of *Ocimum sanctum*, *W. somnifera*, *Emblica officinalis*, *T. cordifolia*, *Mangifera indica*, and *Asphaltum* (*shilajit*) was effective against AIV H9N2 and IBDV coinfection by enhancing antibody levels, thereby increasing survival rate and body weight gain, as well as reducing the viral load in the bursa of Fabricius [24]. More information about herbal plants with antiviral properties is documented in Table-1.

**Table-1:** List of herbal extract that acts as antiviral in poultry.

Plants (species)	Botanical name	Parts used	Mode of action	Virus species	Reference
*A. indica* *C. swynnertonii*	Neem Burtt	Leaves various parts	Reducing the NDV-stimulated splenocyte proliferation	NDV	[15]
*D. crassirhizomatis* *F. mume*	Mianmaguanzhong Dark plum	Rhizoma Fruits	Improved the antibody level and decreased the virus load in the targeted organs.	IBDV	[16]
*C. sinensis*	Green tea	Leaves	Suppressed RNA synthesis by inhibiting the endonuclease activity of RNA polymerase	AIV IBH-HPS	[17,18]
*N. sativa*	Black cumin	Seeds	Stimulated lymphocyte proliferation and immunity development	AIV	[19,20]
Virgin coconut oil	VCO	Dried coconut kernel	Promoted T-lymphocyte production resulting in the improvement of T-helper cells, as well as stimulating antibody production from B-lymphocyte cells.	AIV	[21]
*W. somnifera* T. cordifolia *A. indica*	Ashwagandha Guduchi Neem	Root Stem Leaves	Resist virus multiplication and pathogenesis	CIAV	[22,23]
*O. sanctum* W. somnifera E. officinalis T. cordifolia *M. indica*	Tulsi Ashwagandha Malaka/amla Giloy Mango	Leaves Root Fruit Leaves Fruit	Enhanced the host antibody levels and depressed the viral load in bursa of Fabricius	AIV H9N2 and IBDV coinfection	[24]

NDV=Newcastle disease virus; IBDV=Infectious bursal disease; AIV=Avian influenza virus; IBH-HPS=Inclusion body hepatitis-hydropericardium syndrome; CIAV=Chicken infectious anemia virus. *C. sinensis=Camellia sinensis, W. somnifera=Withania somnifera, T. cordifolia=Tinospora cordifolia, A. indica=Azadirachta indica, O. sanctum=Ocimum sanctum, E. officinalis=Emblica officinalis, M. indica=Mangifera indica, C. swynnertonii=Commiphora swynnertonii, D. crassirhizomatis=Dryopteridis crassirhizomatis, F. mume=Fructus* mume

## Antibacterial

The increasing population of bacteria resistant to antibiotics has threatened the safety of food products, such as chicken, and humans that consume them. Many studies highlight the antibacterial properties of medicinal plants, which are as important as existing synthetic drugs. This potential has become the basis of increasing interest in choosing medicinal plants as an alternative medicine for poultry.

Tannin contained in Amla (*Phyllanthus emblica*) fruit powder [25] can kill bacteria by stimulating phagocytic cells [26]. Amla consumption stimulates increased lactic acid by *Lactobacilli* in the gut, which leads to diminished pH in the intestine to prevent coliforms from merging themselves within the intestinal mucosa and ultimately reduce tissue damage caused by failure from toxin production [27]*. Salvia rosmarinus* (rosemary) supplementation of the broiler diet can increase *Lactobacilli* counts and minimize *Escherichia coli* levels [28]. Similarly, Hanan [29] reported that *Curcuma longa* (turmeric) supplementation mitigated the viability of *E. coli* in the cecum, while increased *Lactobacilli* count affected weight gain.

Lannaon [30] found that herbal combinations of avocado leaves, guava, duhat, *Eucalyptus*, or tamarind trees can be a potential antibiotic for broiler chickens, which performed better than existing antibiotics. Another study by Mapatac [31] showed that administering guava decoction improved performance of broilers among other plants, namely, avocado (*Persea americana*) and malunggay (*Moringa oleifera*) leaves. However, other studies report that consumption of various parts of avocado may show effective antibacterial properties. These leaves and bark extracts contain saponins, tannins, flavonoids, and terpenoids, which are effective against *E. coli* and *Staphylococcus aureus* [32]. Further, the fruit contains defensin PaDef, which is potent in killing *E. coli* and *S. aureus* [33], and the seed contains a high phenolic compound effective against *E. coli*, *S. aureus*, and *Streptococcus agalactiae* [34].

The fruits of guava (*Psidium guajava)* can inhibit the growth of *E. coli* and *Salmonella* Typhimurium [35], while duhat (*Syzygium cumini*) stems contain bioactive compounds, such as flavonoids, tannins, terpenoids, and alkaloids that inhibit the growth of *Bacillus amyloliquefaciens*, *S. aureus*, *E. coli*, and *Pseudomonas aeruginosa* [36]. *Eucalyptus* (*Eucalyptus globulus* L.) leaves contain bioactive compounds, such as tannins, flavonoids, volatile oils, and terpenoids to inhibit the activity of *Salmonella*, *Klebsiella* spp., *S. Streptococcus A*., *Proteus* spp., and *S. aureus* [37]. Tamarind (*Tamarindus indica*) fruit also contains bioactive compounds, such as alkaloids, flavonoids, saponins, and tannins to inhibit the activity of *E. coli*, *Klebsiella pneumoniae*, *Salmonella paratyphi A*., and *P. aeruginosa* [38].

Thyme also delayed the growth of *E. coli* [39] and *S*. Typhimurium [40] *in vitro*. The essential oil of cinnamon (*Cinnamomum zeylanicum*) improved antibacterial properties against foodborne bacteria, such as *Klebsiella* spp., *E. coli*, *Listeria monocytogenes*, and *Bacillus* spp. [41,42]. Moreover, Griggs and Jacob [43] reported that cinnamon (*C. zeylanicum*) essential oil has potential natural antibiotic properties in poultry. In addition, *Allium sativum*, *Allium cepa*, and *Menthe* spp. can effectively reduce the growth of *E. coli* [44]. More information about herbal plants with antibacterial properties is documented in Table-2.

**Table-2:** List of herbal extract that acts as antibacterial in poultry.

Plants (species)	Botanical name	Parts used	Mode of action	Bacteria species	Reference
*P. emblica*	Amla	Fruit	Stimulating phagocytic cells, Increased *Lactobacilli* population in gut, Failure of bacterial colonization in intestine	*Coliforms*	[25-27]
*S. rosmarinus*	Rosemary	Leaves	Increased the *Lactobacill*i counts and minimized *E. coli level*	*E. coli*	[28]
*C. longa*	Turmeric	Rhizome	Increased the *Lactobacilli* counts and minimized *E. coli* level	*E. coli*	[29]
*P. americana* Mill	Avocado	Leaves and bark	Role of bioactive compounds such as saponins, tannins, flavonoids, and terpenoids	*E. coli, S. aureus*	[32]
		Fruit	Defensin PaDef as antimicrobial peptides	*E. coli, S. aureus*	[33]
		Seed	Phenolic compounds as well documented antimicrobial	*E. coli, S. aureus*, *S. agalactiae*	[34]
*P. guajava*	Guava	Fruit	Inhibit the growth of bacteria	*E. coli, S.* Typhimurium	[35]
*S. cumini*	Duhat	Stem	The bioactive such as flavonoids, tannin, terpenoid, and alkaloid inhibits the growth of bacteria	*B. amyloliquefaciens, S. aureus, E. coli*, *P. aeruginosa*	[36]
*E. globulus* L.	Eucalyptus	Leaves	The bioactive properties such as tannin, flavonoids, volatile oils, and terpenoids inhibit bacterial activity	*Salmonella, Klebsiella* spp., *S. Streptococcus A., Proteus* spp., *S. aureus*	[37]
*T. indica*	Tamarind	Fruits	The bioactive properties such as alkaloid, flavonoid, saponin, and tannin inhibit bacterial activity	*E. coli, K. pneumoniae, S. paratyphi A., P. aeruginosa.*	[38]
*T. vulgaris*	Thyme	Leaves	Delayed the growth of bacteria	*E. coli, S*. Typhimurium	[39, 40]
*C. zeylanicum*	Cinnamon	Inner bark	Improved the antibacterial properties against foodborne bacteria	*Klebsiella* spp., *E. coli, L. monocytogenes,* and *Bacillus* spp.	[41, 42, 43]
*A. sativum* A. cepa *Menthe* spp.	Garlic Mint Onion	Bulbs Leaves Leaves	Inhibit the growth of bacteria	*E. coli*	[44]

*S. rosmarinus*=*Salvia rosmarinus*, *E. coli*=*Escherichia coli*, *C. longa*=*Curcuma longa*, *P. americana*=*Persea americana, S. aureus*=*Staphylococcus aureus*, *S. agalactiae*=*Streptococcus agalactiae*, *P. guajava*=*Psidium guajava, Salmonella* Typhimurium: *S.* Typhimurium, *S. cumini*=*Syzygium cumini, B. amyloliquefaciens*=*Bacillus amyloliquefaciens, P. aeruginosa*=*Pseudomonas aeruginosa*, *E. globulus*=*Eucalyptus globulus*, *T. indica*=*Tamarindus indica*, *K. pneumonia=Klebsiella pneumonia, S. paratyphi A=Salmonella paratyphi A*, *C. zeylanicum*=*Cinnamomum zeylanicum, L. monocytogenes*=*Listeria monocytogenes, A. sativum*=*Allium sativum, A. cepa*=*Allium cepa, T. vulgaris*=*Thymus vulgaris*, *P. emblica*=*Phyllanthus emblica*

## Antifungal

*Aspergillus fumigatus* causes frequent respiratory disease in birds called aspergillosis compared with other species, such as *Aspergillus flavus*, *Aspergillus niger*, *Aspergillus Nidulans*, and *Aspergillus terreus* [45]. Plant extracts contain phenolic compounds as potential antifungal and antiaflatoxigenic agents [46] using various mechanisms, such as inhibiting the production of aflatoxin B1, namely, syringaldehyde, sinapic acid, and acetosyringone [47], and decreasing *A. flavus* growth by targeting oxidative mitochondrial stress as a defense system using substances such as salicylic acid, thymol, vanillyl acetone, cinnamic acid, and vanillin [48].

Njoki *et al*. [49] showed that *Spinacia oleracea* extracts were effective antifungals by diminishing the conidia of toxigenic *A. flavus*. The extracts had 19.6% saponins, flavonoids, terpenoids, tannins, alkaloids, and glycosides and the antifungal properties of the extracts of *Artemisia dracunculus*, *Achillea wilhelmsii*, *Bunium persicum*, *C. cyminum*, *Zataria multiflora* Boiss, and *Satureja hortensis* were studied against *A. niger*, *A. fumigatus*, *A. flavus* [50], and *Candida albicans* [51]. Among the extracts tested, *A. wilhelmsii* and *Z. multiflora* Boiss were the most effective antifungals. Plant extracts contained phenolic isomers, namely, thymol and carvacrol.

Furthermore, other avian pathogenic fungi, such as *Alternaria* spp., were successfully inhibited by *Salvia sclarea*, *Salvia officinalis*, and *Rosmarinus officinalis* extracts [52]. The fungistatic and fungicidal mechanisms involved synthesizing bioactive organic compounds [53], as well as antifungal protein [54] and peptide [55].

The seeds and fruit extract of *Carum copticum* can be effective against *Aspergillus* spp. by inhibiting growth [56]. Further, leaf extract of *Satureja bachtiarica* contains thymol and g-terpinene that can limit the growth of *C. albicans*, *Candida krusei*, and *Kennedia glabrata* [57]. The combination of *Prosopis spicigera*, *Zingiber officinale*, and *Trachyspermum ammi* are rich in alkaloids, amino acids, protein, sterols, and terpenes that are toxic toward *C. albicans*, *C. krusei*, *C. tropicalis*, and *Candida glabrata* [58]. The aromatic ring and the presence of free phenol hydroxyl of *Mentha piperita* and *Thymus vulgaris* L. formulation activity can damage fungal cell walls and membranes, such as *Aspergillus*, *Penicillium* spp., *Fusarium*, and *Saccharomyces* [59].

Similarly, oil extracts of *Z. multiflora* Boiss, *Cinnamomum verum*, and *Pimpinella anisum* can inhibit the growth of *C. albicans*, *A. flavus*, *A. niger*, and *A. fumigatus* [60]. The combination of *Mentha arvensis*, *O. sanctum*, and *A. sativum* was potent in preventing the growth of mycelial cells of *A. fumigatus*, *Aspergillus parasiticus*, and *Penicillium* spp. [61]. Allicin content of *Allium tuncelianum* is a toxigenic agent against *C. albicans* and *Malassezia pachydermatis* [62]. Similarly, the production of aflatoxin B1 of *A. flavus* was stopped by *C. longa* L. [63]. Further herbal extract studies on antifungal properties in poultry are listed in Table-3.

**Table-3:** List of herbal extract that acts as antifungal in poultry.

Plants (species)	Botanical name	Parts used	Mode of action	Fungi species	Reference
*S. oleracea*	Spinach	Leaves	Diminishing the conidia of toxigenic fungus.	*A. flavus*	[49]
*A. wilhelmsii*, *Z. multiflora Boiss*	Yarrow, Shirazi thyme	Flower	Inhibit the growth of fungus	*A. niger, A. fumigatus,* and *A. flavus* *Candida albicans*	[50] [51]
*S. sclarea* S. officinalis *R. officinalis*	Clary sage Sage Rosemary	Leaves	Synthesis of the compounds of bioactive organic, also antifungal protein and peptide	*Alternaria* spp.	[52-55]
*C. copticum*	Ajwain	Seeds, fruits	Inhibit the growth of fungus	*Aspergillus* spp.	[56]
*S. bachtiarica*	Savory	Leaves	Thymol and g-terpinene are able to inhibit the growth of fungus	*C. albicans, C. krusei, K. glabrata*	[57]
*P. spicigera* Z. officinale *T. ammi*	Khejri Ginger Ajwain	Leaves Roots Seeds	The phytoconstituents such as alkaloids, amino acids, protein, sterols, and terpenes	*C. albicans, C. krusei, C. tropicalis, C. glabrata*	[58]
*M. piperita* *T. vulgaris* L.	Peppermint thyme	Oil	The aromatic ring and the presence of the free phenol hydroxyl activity leads to the damage of fungal cell wall and cell membrane	*Aspergillus, Penicillium* spp., *Fusarium, Saccharomyces*	[59]
*Z. multiflora Boiss* C. verum *P. anisum*	Thyme Cinnamon Anise	Oil	Inhibit the growth of isolated fungal	*C. albicans, A. flavus, A. niger. A. fumigatus*	[60]
*M. arvensis* O. sanctum *A. sativum*	Mint Tulsi Garlic	Leaves Leaves Bulbs	Inhibit the mycelial growth of fungus	*A. fumigatus, A. parasiticus, Penicillium* spp.	[61]
*A. tuncelianum*	Tunceli garlic	Bulbs	The allicin substance is able to inhibit the growth of fungus	*C. albicans, M. pachydermatis*	[62]
*C. longa L.*	Turmeric	Rhizomes	Inhibit fungus activity and prevent the production of Aflatoxin B1	*A. flavus*	[63]

*C. longa=Curcuma longa, A. sativum*=*Allium sativum*, *A. fumigatus*=*Aspergillus fumigatus, A. flavus*=*Aspergillus flavus, A. niger*=*Aspergillus niger, S. oleracea*=*Spinacia oleracea, A. wilhelmsii*=*Achillea wilhelmsii, S. sclarea*=*Salvia sclarea, S. officinalis*=*Salvia officinalis, R. officinalis=Rosmarinus officinalis, C. copticum*=*Carum copticum, C. krusei=Candida krusei, C. glabrata*=*Candida glabrata, K. glabrata*=*Kennedia glabrata, P. spicigera*=*Prosopis spicigera, T. ammi=Trachyspermum ammi, M. piperita=Mentha piperita, T. vulgaris*=*Thymus vulgaris, C. verum*=*Cinnamomum verum, P. anisum=Pimpinella anisum, M. arvensis=Mentha arvensis*, *A. parasiticus: Aspergillus parasiticus, M. pachydermatis*=*Malassezia pachydermatis, S. bachtiarica=Satureja bachtiarica, A. tuncelianum*=*Allium tuncelianum, Z. officinale*=*Zingiber officinale*

## Antiparasitic

### Antiprotozoal

Antiprotozoal works by destroying the protozoa itself or inhibiting their growth and reproductive capabilities. Some commercial drugs, such as mebendazole, metronidazole, nitroimidazole dimetridazole, ornidazole, tinidazole, monensin, salinomycin, and semduramycin, have been regulated in many countries. Since then, diseases caused by protozoa has increased and resulted in economic losses.

Habibi *et al*. [64] observed that consumption of *Nectaroscordum tripedale* with diclazuril leads to improved growth performance, lesion score, the extent of bloody diarrhea, and oocyst count in individuals infected by *Eimeria tenella*. The herb can promote body mass gain and has previously been shown to alleviate lesion scores in birds compared with the control group.

Muthamilselvan *et al*. [65] evaluated other anticoccidial herbs, including *Artemisia annua*, pine bark, *A. sativum*, and *C. sinensis*. *A. annua* contains artemisinin that can stimulate reactive oxygen species to inhibit *E. tenella* sporulation and cell wall formation [66]. Further, sulfur content of *A. sativum* is effective in preventing *E. tenella* sporulation. Pine bark extract contains condensed tannins that influx the wall of oocytes, damage the cytoplasm, and inactivate endogenous enzymes responsible for the sporulation mechanism. Sporocytes in oocytes then turn abnormal and lead to diminished sporulation of oocytes in *E. tenella*, *Eimeria Maxima*, and *Eimeria acervulina* [67]. Selenium and polyphenol substances of *C. sinensis* are also effective anti-coccidiosis agents leveraging the same mechanism [68]. Further, previous reports show that allicin of *A. sativum* can effectively inhibit the sporulation of *E. tenella* [69]. N-3 fatty acids, flavonoids, and vernoside extracted from *Linum usitatissimum* [70], *Ageratum conyzoides* [71], and *Vernonia amygdalina* [72] are also potential herbs for the same parasites. A previous study reported that *Carica papaya* (*papain*) leaves [73] and saponin and betaine source plants (*Cyamopsis tetragonoloba*, *Mesembryanthemum cordifolium*, *Morinda citrifolia*, and *Malvaviscus arboreus*) lysed oocysts and protected the epithelial cells [74]. The essential oils of *Origanum vulgare*, olive leave extract, grape seed extract, guar, *T. vulgaris*, turmeric, and clove extract are also effective in destroying parasites [75].

The coccidiostat effect was also studied by Christaki *et al*. [76] who extracted apacox from the combination of *Agrimonia eupatoria*, *Echinacea angustifolia*, *Ribes nigrum*, and *Cinchona succirubra*, showing a coccidiostatic effect on *E. tenella*, despite the effect being significantly lower than the control group. Furthermore, Gefu *et al*. [77] showed that onion (*A. cepa*) is a potential antiparasitic against protozoa, such as *Leishmania* spp. The parasite was successfully eradicated after onion extract was administered at a 1.25 mg/mL dose [78]. The pronounced antiprotozoal activity of onion is due to the sulfur compound alliin and its ability to treat lesions caused by protozoa [79].

*Histomonas meleagridis* is protozoa causing high mortality and morbidity in poultry. A study by Hafez and Hauck [80] reported that essential oils combining *C. verum*, *A. sativum*, *Citrus limon*, and *S. rosmarinus* provide prophylactic effects that can diminish mortality in infected animals. The herbal properties as an antiprotozoal agent in poultry are summarized in Table-4.

**Table-4:** List of herbal extract that acts as antiprotozoal in poultry.

Plants (species)	Botanical name	Parts used	Mode of action	Protozoa species	Reference
*N. tripedale* with diclazuril	Sicilian honey garlic	Bulbs	*Improved* growth performance, lesion score, extent of bloody diarrhea, and oocyst count in infected group by protozoa	*E. tenella*	[64]
*A. annua*	Artemisinin	Leaves	Inhibit the sporulation of protozoa	*E. tenella*	[65,66]
*P. radiata*	Pine	Bark		*E. tenella, E. acervuline, A. maxima*	[67]
*C. sinensis*	Tea plant	Leaves		*E. tenella, E. acervuline, A. maxima*	[68]
*A. sativum*	Garlic	Bulb		*E. tenella, E. acervuline, A. maxima*	[69]
*L. usitatissimum*	Flax	Seed	*N-3 fatty acids* suppressed the development in schizogony stage	*E. tenella*	[70]
*A. conyzoides*	Billy goat weed	Leaves	Flavonoids suppressed the development in schizogony stage	*E. tenella*	[71]
*V. amygdalina*	Delile	Leaves	Inhibit the sporulation of oocysts	*E. tenella*	[72]
*C. papaya*	Papain	Leaves	Stimulate host immunity and disturb the oocyst formation	*E. tenella*	[73]
*C. tetragonoloba*	Guar	Bean		*E. tenella*	[74]
*M. cordifolia*	Baby sun rose	Leaves	Lysed the oocysts and protected the epithelial cells	*E. tenella*	[74]
*M. citrifolia*	Noni	Fruits	Lysed the oocysts and protected the epithelial cells	*E. tenella*	[74]
*M. arboreus*	Wax mallow	Leaves	Lysed the oocysts and protected the epithelial cells	*E. tenella*	[74]
			Lysed the oocysts and protected the epithelial cells	*E. tenella*	[74]
*O. vulgare*	Oregano	Dried leaves, essential oil	Destruction of sporozoite membrane and suppression of oocyst production	Various species	[75]
*O. europaea*	Olive leave	Leaves	Anti-inflammatory and anti	*E. tenella*	[75]
*V. vinifera*	Grape	Seed	Oxidative stress	*E. tenella*	[75]
*C. tetragonoloba*	Guar	Bean	Damage the cell membrane	*E. tenella*	[75]
*T. vulgaris*	Thyme	Leaves	Destroy the oocysts	*E. tenella*	[75]
*C. longa L.*	Turmeric	Rhizomes	Damage the sporozoite membranes	*E. tenella*	[75]
*S. aromaticum*	Clove	Leaves	Damage the oocytes	*E. tenella*	[75]
*A. eupatoria*	Agrimony	Leaves	Enhancing body weight gain and value of feed conversion ratio	*E. tenella*	[76]
*E. angustifolia*	Purple coneflower	Petals	Enhancing body weight gain and value of feed conversion ratio	*E. tenella*	[76]
*R. nigrum*	Blackcurrant	Seed, leaves, fruit	Enhancing body weight gain and value of feed conversion ratio	*E. tenella*	[76]
*C. succirubra*	Quina	Bark	Enhancing body weight gain and value of feed conversion ratio	*E. tenella*	[76]
*A. cepa*	Onion	Bulbs	The sulfur compound allicin is able to eradicate the protozoa and its ability to treat the lesion caused by the protozoa	*Leishmania* spp.	[77, 78, 79]
*C. verum* A. sativum C. limon *S. rosmarinus*	Cinnamon Garlic Lemon Rosemary	Oils	The essential oils contain prophylactic effect that able to diminish mortality in infected animals	*H. meleagridis*	[80]

*C. sinensis=Camellia sinensis*, *S. rosmarinus=Salvia rosmarinus*, *C. longa=Curcuma longa*, *A. sativum=Allium sativum*, *A. cepa=Allium cepa, T. vulgaris=Thymus vulgaris*, *C. verum=Cinnamomum verum*, *E. tenella=Eimeria tenella*, *A. annua=Artemisia annua*, *L. usitatissimum=Linum usitatissimum, A*. *conyzoides=Ageratum conyzoides*, *V. amygdalina=Vernonia amygdalina*, *C. tetragonoloba=Cyamopsis tetragonoloba, M. citrifolia=Morinda citrifolia, M. arboreus=Malvaviscus arboreus*, *O. vulgare=Origanum vulgare*, *A. eupatoria=Agrimonia eupatoria*, *E. angustifolia=Echinacea angustifolia*, *R. nigrum=Ribes nigrum*, *C. succirubra=Cinchona succirubra*, *H. meleagridis=Histomonas meleagridis*, *C. limon=Citrus limon, P. radiate=Pinus radiate*, *V. vinifera=Vitis vinifera*, *S. aromaticum=Syzygium aromaticum*

### Anthelmintic

The residual effects on chicken meat or eggs due to drug consumption and reducing synthetic drug use in chickens are pertinent issue, which not only causes resistance but also incurs additional residual impacts that endanger humans and the environment. Pumpkin oil (*Cucurbita pepo* L.) acts as an anthelmintic, as well as a natural laxative, by reducing helminth count and egg output [81,82]. Consumption of myrrh and thyme can also kill helminths, such as *Trichinella spiralis* [83]. Administering onion oil at a concentration of 5 mg/kg/day is potent on mature worms and the cystic stage of *T. spiralis*, which can also enhance protective antibodies against the parasite [84]. In 2003, Zenner *et al*. [85] studied the influence of cinnamon oil on *Trichomonas* and *H. meleagridis* [85]. The oil contains trans-cinnamaldehyde and proanthocyanidins that promote its anthelmintic effects [86], disrupting the intestinal tissue of the parasites.

Both *in vitro* and *in vivo* studies have explored the anthelmintic activity of leaf extracts of *Tephrosia vogelii* and *V. amygdalina* against *A. galli* infections. The plant extracts comprise chemical elements, such as rotenoids, lactones, sesquiterpene, glycosides, tannins, and anthracenes, which all showed significant anthelmintic activity (p<0.05) against *A. galli*. The *in vitro* study demonstrated that both extracts effectively inhibited 74.7% and 63.7% of larval migration, respectively, while fecal egg counts decreased by 77.4% and 76.9%, respectively [87]. Other plants also target the same helminth. Papaya (*C. papaya*) extracts are effective anthelmintics against the growth of *A. galli* eggs due to proteolytic enzymes, such as papain, chymopapain, and lysozymes found in the latex, as well as the leaves [88]. Here, papain inhibited all developmental stages of the parasite. The study also suggested that the dust of bishkatali leaves can inhibit the development of *A. galli* eggs. The table below provides detailed information on various plants that act as anthelmintics in poultry (Table-5).

**Table-5:** List of herbal extract that acts as anthelmintic in poultry.

Plants (species)	Botanical name	Parts used	Mode of action	Helminths species	References
*C. pepo* L.	Pumpkin	Seeds	Decrease the worm count and eggs output of the worm	*Ascaridia spp., Raillietina* spp., *Heterakis* spp.	[81,82]
*C. myrrha* and *T. vulgaris*	Myrrh and thyme	Resin of the bark, leaves	Decrease the worm count	*T. spiralis*	[83]
*N. sativa* and *A. cepa*	Onion	Bulb extract oil	Potent on the mature worms and cystic stage of the worm, also improving the protective antibodies against the parasite	*T. spiralis*	[84]
*C. verum*	Cinnamon	Oil	The oil contains trans-cinnamaldehyde and proanthocyanidins disrupting the intestinal tissue of the parasites	*Trichomonas* and *H. meleagridis*	[85]
*T. vogelii* and *V. amygdalina*	Vogel *Tephrosia* and Delile	Leaves	The plant extracts consist of chemical elements such as rotenoids, lactones, sesquiterpene, glycosides, tannins, and anthracenes, which all showed significant anthelmintic activity against the worm. Inhibited larval migration, while fecal egg counts were reduced	*A. galli*	[86]
*C. papaya*	Papaya	Latex and leaves	Suppress the growth of the worm and inhibit all developmental stages of the parasite due to proteolytic enzymes such as papain, chymopapain, and lysozymes	*A. galli*	[87]
*Pale persicaria*	Bishkatali	Leaves	Inhibit the development of the eggs of the worm	*A. galli*	[88]

*N. sativa*=*Nigella sativa*, *A. cepa=Allium cepa*, *T. vulgaris=Thymus vulgaris*, *C. verum=Cinnamomum verum*, *V. amygdalina=Vernonia amygdalina*, *C. papaya=Carica papaya*, *H. meleagridis=Histomonas meleagridis*, *C*. *pepo=Cucurbita pepo*, *T. spiralis=Trichinella spiralis*, *T. vogelii=Tephrosia vogelii*, *C. myrrha=Commiphora myrrha*

### Lice and mice

The use of chemical insecticides may harm birds since the toxic effects directly jeopardize birds and possibly contaminate chicken meat. Jacob and Pescatore [89] proved that the aqueous extract of garlic and cinnamon oil kills lice and mice infestations in chickens, including *Trichomonas*, *H. meleagridis*, and head lice. Other herbal products effective against lice infestation in poultry include 2% aqueous solution of Pestoban [90] at a 1:30 dilution [91]. Another study showed that garlic extracts killed 96.5% of *Dermanyssus gallinae* infestation in layer farms [92] and mites show overall body damage under scanning electron microscope examination, including darkened leg coloring. Unfortunately, no further information exists on histopathological changes to mites induced by herbs. Pumnuan *et al*. [93] suggested that clove essential oil is a natural insecticide to control chicken lice (*Lipeurus caponis* L.), while spices such as *Annona senegalensis*, *Tectona grandis*, *Securidaca longepedunculata*, *Indigofera hirsuta*, *Lophira lanceolata*, *Hyptis spicigera*, *Steganotaenia araliacea*, *Oxytenanthera abyssinica*, *Nicotiana tabacum*, *Jatropha curcas*, *Ficus exasperata*, *A. indica*, and *Parkia biglobosa* are effective insecticides, with *A. senegalensis* being the most popular endogenous plant in controlling ectoparasites in backyard poultry [94]. Table-6 describes herbs having anti-ectoparasite properties for poultry (Table-6).

**Table-6:** List of herbal extract that acts as anti-ectoparasites in poultry.

Plants (species)	Botanical name	Parts used	Mode of action	Ectoparasites species	References
*A. sativum* and *C. verum*	Garlic and cinnamon oil	Bulbs and bark	Eliminate lice and mice infestations in chickens	*Trichomonas*, *H. meleagridis,* and head lice	[89]
*C. deodara, A. indica, E. ribes*	Pestoban	Fruit	Eliminate lice and mice infestations in chickens	*D. gallinae, Knemidocoptes mutans*	[90,91]
*A. sativum*	Garlic	Bulbs	Eliminate lice and mice infestations in chickens	*D. gallinae*	[92]
*S. aromaticum*	Clove	Flower buds	Eliminate lice and mice infestations in chickens	*L. caponis* L.	[93]
*A. senegalensis*	African custard- apple	Fruit	Eliminate lice and mice infestations in chickens	*D. gallinae*	[94]
*T. grandis*	Teak	Flower			
*S. longepedunculata*	Violet tree	Leaves			
*I. hirsuta*	Hairy indigo	Leaves			
*L. lanceolata*	Dwarf red ironwood	Seeds			
*H. spicigera*	Black sesame	Seeds			
*S. araliacea*	Apiaceae	Leaves			
*O. abyssinica*	(A. Rich) Munro	Seeds			
*N. tabacum*	Tobacco	Leaves			
*J. curcas*	Jarak pagar	Leaves			
*F. exasperata*	Moraceae	Leaves			
*A. indica*	Neem	Leaves			
*P. biglobosa*	African locust bean tree	Pods			

*A. indica=Azadirachta indica*, *A. sativum=Allium sativum*, *C. verum=Cinnamomum verum*, *H. meleagridis=Histomonas meleagridis*, *D. gallinae=Dermanyssus gallinae*, *L. caponis=Lipeurus caponis*, *A. senegalensis=Annona senegalensis*, *T. grandis=Tectona grandis, S. longepedunculata=Securidaca longepedunculata, I. hirsute=Indigofera hirsute, L. lanceolata=Lophira lanceolata*, *H. spicigera=Hyptis spicigera*, *S. araliacea=Steganotaenia araliacea*, *O. abyssinica=Oxytenanthera abyssinica, N. tabacum=Nicotiana tabacum, J. curcas=Jatropha curcas, P. biglobosa=Parkia biglobosa*, *C. deodara=Cedrus deodara*, *E. ribes=Embelia ribes*, *S. aromaticum=Syzygium aromaticum*, *F. exasperata=Ficus exasperata*

## Immunomodulator

Medicinal plants contain compounds to stimulate immunity. Plant extracts have been recommended as immunomodulators due to their effects on the animal immune system related to phytochemicals, such as flavonoids, polysaccharides, lactones, alkaloids, diterpenoids, and glycosides. Many medicinal plants show less toxic side effects; hence, they are considered a safe treatment compared to synthetic therapy.

In poultry, plant extracts containing a mixture of cinnamaldehyde, carvacrol, and capsicum oleoresin as immunomodulators, showed regression of CD_40_ LG, interferon (IFN)-G, and interleukin (IL)-6, indicating low inflammation, especially in the digestive tracts against avian coccidiosis caused by intestinal protozoan parasites and *Eimeria* spp. [95]. The overall effects were confirmed by higher levels of serum antibodies and enhanced pro-inflammatory cytokine production in the duodenum.

Another study on the role of *Astragalus membranaceus* as an immunomodulator showed improved immune activity in broiler chicks infected with lipopolysaccharide [96]. The study showed that the immune organ weight and IgG level increased, while liver and kidney functions improved. The plant also stimulated the growth of fecal microorganism composition. Tan and Vanitha [97] reported that *Aloe vera* leaves contain CARN750 that selectively stimulates immunomodulatory substances, such as cytokine and lymphocytes, while the roots and leaves of *Panax ginseng* are rich in saponin can effectively stimulate lymphocytes, cytokine, and IL-6 to improve macrophage performance. The herbs offered potential resistance to viral infections in birds by stimulating macrophage production, as well as T-cell-mediated immune responses and stimulating macrophages through TLR6 signaling and NF kappa B translocation, leading to cytokine production. The hematological profile also improved after synthesizing more hemoglobin in the bone marrow after hepatic cells released erythropoietic factors. In a study of 42-day-old broilers given sheep red blood cells, turmeric rhizome powder improved blood immunoglobulin A, immunoglobulin G, and immunoglobulin M levels, and minimized the monocyte ratio [98]. However, insufficient doses may fail to improve immune response [99]. Tamam *et al*. [100], and Das *et al*. [101] reported that *C. verum* essential oil promotes macrophages, phagocytosis, and killing of invading microorganisms by macrophages, facilitating the body’s primary line of defense against Newcastle disease infection. The table below shows herbs that act as immunomodulators in poultry (Table-7).

**Table-7:** List of herbal extract that acts as immunomodulator in poultry.

Plants (species)	Botanical name	Parts used	Mode of action	Agent	References
*Capsicum* L.	Capsicum	Fruits	Regression of CD_40_LG, IFN-G, and IL-6, indicating low inflammation, higher levels of serum antibodies and enhanced pro-inflammatory cytokine production in the duodenum	*Eimeria* spp.	[95]
*A. membranaceus*	Huangqi	Leaves, roots	Improved immune activity in broiler chicks infected with lipopolysaccharide The immune organ weight and IgG level increased, while the liver and kidney functions improved. The plant stimulated the growth of the fecal microorganism composition	Unspecified	[96]
*A. vera* *P. ginseng*	Aloe Asian ginseng	Leaves Roots and leaves	CARN750 compound stimulate cytokine and lymphocytes, while the roots and leaves of *P. ginseng* rich in saponin that effectively stimulate lymphocytes, cytokine, and IL-6, and improve the work of macrophage. potential resistance to viral infections in birds by stimulating macrophage production as well as T-cell-mediated immune responses, stimulating macrophages through TLR6 signaling and NF-kappa B. Improved hematological profile	Unspecified	[97]
*C. longa*	Turmeric	Rhizome powder	Improved blood IgA, IgG, and IgM levels, and minimized the monocyte ratio	Unspecified	[98,99]
*C. verum*	Cinnamon	Barks	Promotes macrophages, phagocytosis, and killing of invading microorganisms by macrophages, facilitating the body’s primary line of defense against viral infection	NDV	[100]

NDV=Newcastle disease virus, IL=Interleukin, INF=Interferon *C. longa=Curcuma longa*, *C. verum=Cinnamomum verum*, *A. membranaceus=Astragalus membranaceus*, *P. ginseng=Panax ginseng*, *A. vera=Aloe vera*

## Antioxidants

Adding *Borreria latifolia* to the chicken diet can help prevent lipid peroxidation [102]. The phenols act to minimize lipid peroxidation and inhibit free radicals and oxidative deterioration, which can improve chicken meat quality. Antioxidant properties have also been observed in broiler chicken meal supplemented with a mixture of bitter leaf meal and *M. oleifera* leaf without affecting serum biochemistry of the experimental birds [103]. Both leaves contain phytochemicals, such as flavonoid, quercetin, and phenol that can increase serum antioxidant enzymes, such as glutathione, catalase, and superoxide dismutases, and decrease lipid peroxidation. A review by Ali *et al*. [104] also mentioned that cinnamon was safe for poultry health and had positive environmental and economic aspects. Bravo *et al*. [105] demonstrated increased energy utilization and growth performance in broilers after administering a mixture of cinnamaldehyde, carvacrol, and capsicum oleoresin. The additive effect of plants supports antioxidant enzyme function of the mucosal layer cells that protect the tissue [106], suggesting the potential for plant extracts as antioxidants. The table shows herbs that act as antioxidants for poultry (Table-8).

**Table-8:** List of herbal extract that acts as antioxidant in poultry.

Plants (species)	Botanical name	Parts used	Mode of action	Reference
*B. latifolia*	Broadleaf button weed	Leaves	The phenols act to minimize lipid peroxidation to inhibit free radicals and oxidative deterioration which improves the chicken meat quality	[102]
*V. amygdalina* and *M. oleifera*	Bitter leaf meal and drumstick leaf	Leaves	Contain phytochemicals such as flavonoid, quercetin, and phenol that increased serum antioxidant enzymes such as GSH, catalase, and SOD and decreased lipid peroxidation	[103]
*C. verum*	Cinnamon	Barks	Support anti-inflammatory effect	[104]
*Capsicum* L.	Capsicum	Fruits	Increased energy utilization and growth performance in broilers Supports the function of antioxidant enzymes of the mucosal layer cells that protect the tissue	[105,106]

SOD=Superoxide dismutase, *M. oleifera=Moringa oleifera*, *C. verum=Cinnamomum verum*, *V. amygdalina=Vernonia amygdalina*, *B. latifolia=Borreria latifolia*

## Body Weight Gain

Administering medicinal plants in animal production health are increasing following several studies that have highlighted the additive effects of plants in improving biological development. Herbal medicines, such as white turmeric (curcumin), red ginger (zingerone), galangal (methyl-cinnamic), and garlic (allicin), are alternative treatments due to their ability to increase the viability of chickens, as indicated by a decrease in the percentage mortality and body weight gain. These plants improved digestibility and durability of treated chickens, resulting in better nutrient intake [107]. Further, a combination of *A. sativum*, *Urtica dioica*, *Inula helenium*, *Glycyrrhiza glabra*, *R. officinalis*, *Chelidonium majus*, *Thymus serpyllum*, *Tanacetum vulgare*, and *Coriandrum sativum* increased chicken weight gain and food conversion ratio in an anti-coccidial study [108]. This formula is rich in polyphenols that reduce the oocyst output and decrease the total lesion score, thereby improving digestibility. Naderi *et al*. [109] also revealed that adding 2.5 g of turmeric powder to 1 kg poultry feed significantly increased body weight gain during the starter (0-21 days) period. Including 5 g of *C. longa* powder in a 1 kg diet also improved growth rates of broilers. Turmeric not only increased the weight of bursa of Fabricius but also promoted the production and secretion of bile and digestive enzymes, thereby improving digestion and absorption of dietary nutrients.

Another herb with the same potential is *Allium hookeri* root [110], which contains organosulfur compounds, proteins, volatile sulfur, prostaglandins, fructans, vitamins, and polyphenols that improved body weight gain after 1% *A. hookeri* root was administered on treatment day 14 to broilers. Further, *A. vera* polysaccharides administered to chickens infected with *Eimeria* spp. affected intestinal microflora and reduced intestinal lesions, promoting weight gain, and better performance [111]. Curcumin and turmeric can enhance body weight gain due to their effects on feed intake and improved growth [112]. Dosoky and Setzer [113] showed that curcumin acts as a stimulant, carminative, stimulates digestibility, has antimicrobial properties, and is a gastric toxicity inhibitor, while turmeric (1%) acts as an antioxidant in promoting positive feed conversion ratio [114].

*Moringa stenopetala* leaf meal exhibits high pepsin soluble nitrogen (82-91%), low acid detergent-insoluble protein (1-2%), and low antinutritional factors [115]. It may also contain Vitamin E [116] to improve intestinal microarchitecture and produce acidic mucin, as shown in treated chickens [117]. Cinnamon and thyme administration in a dose of 0.5%, 1% thyme, and 1% cinnamon in chickens with colibacillosis effectively decreased total aerobic bacterial count of the gastrointestinal tract and improved body performance in chickens [118]. These herbs altered cell wall permeability of bacteria and created a pore that leads to osmotic shock and leakage of the cytoplasm, resulting in cell death [119]. Another study showed that nettle or lemon balm can improve chicken body weight after 21 days of administration [120]. Nettle has anti-inflammatory properties and high flavonoid content that relaxes smooth muscles of the digestive tract and bile ducts [121]. It is also rich in antioxidants that act intracellularly [122], which could be responsible for better animal performance. The study also revealed that herbs successfully replaced antibiotic growth promoters; moreover, adding 0.1%, 0.25%, and 0.5% crude preparation of fenugreek, ginger, and turmeric in the diet-stimulated growth rates of gut layers by significantly reducing the excretion of fecal anaerobic bacteria while maintaining excretion of beneficial bacteria [123]. Abdullahi *et al*. [124] revealed that herbs stimulated the production of IL-2, IL-10, tumor necrosis factor, and IFN-g, improving Ig antibody levels in serum, thereby promoting daily weight gain. The table below describes various herbs that improve body weight promoters in poultry (Table-9).

**Table-9:** List of herbal extract that acts as body weight promoter in poultry.

Plants (species)	Botanical name	Parts used	Mode of action	Reference
*C. zedoaria*	White turmeric	Rhizomes	Improved the digestibility and durability of treated chicken, resulting in a better intake of nutrients	[107]
*A. purpurata*	Red ginger	Rhizomes		
*K. galanga*	Galangal	Rhizomes		
*A. sativum*	Garlic	Bulbs		
*A. sativum*	Garlic	Bulbs	The formula is rich in polyphenols that reduce the oocyst output and decrease the total lesion score, thereby improving digestibility	[108]
*U. dioica*	Nettle	Leaves		
*I. helenium*	Elecampane	Rhizomes		
*G. glabra*	Liquorice	Roots		
*R. officinalis*	Rosemary	Leaves		
*C. majus*	Greater celandine	Aerial parts and roots		
*T. serpyllum*	Breckland thyme Tansy	Leaves		
*T. vulgare*				
*C. sativum*	Coriander	Leaves, aerial parts, Seeds, leaves, roots		
*C. longa*	Turmeric	Rhizomes	Increased the weight of bursa of Fabricius but also promoted the production and secretion of bile and digestive enzyme, thereby improving digestion and the absorption of dietary nutrients	[109]
*A. hookeri*	Garlic chives	Roots	The organosulfur compounds, proteins, volatile sulfur, compounds, prostaglandins, fructans, vitamins, and polyphenols that improved body weight gain	[110]
*A. vera*	Aloe	Leaves	The polysaccharides affected the intestinal microflora and reduced intestine lesion, promoting weight gain and better performance	[111]
*C. longa* and *C. aromatica*	Curcumin and turmeric	Rhizomes	Curcumin is a stimulant, carminative, stimulates digestibility, has antimicrobial properties, and is a gastric toxicity inhibitor, while turmeric (1%) acts as an antioxidant in promoting feed conversion ratio	[112-114]
*M. stenopetala*	Cabbage tree	Leaves	The bioactive compounds improved the intestinal microarchitecture and the production of acidic mucin	[115-117]
*C. verum and T. vulgaris*	Cinnamon and thyme	Barks and leaves	Decreased the total aerobic bacteria count of the gastrointestinal tract and improved the body performance of the chicken	[118,119]
*U. dioica*	Nettle	Fruits	Nettle has anti-inflammatory properties and high flavonoid content that relaxed smooth muscles of the digestive tract and the bile ducts. It is also rich in antioxidants that act intracellularly that might be responsible for better animal performance	[120-122]
*T. foenum-graecum*	Fenugreek	Seeds	Stimulated the growth rate of layers by significantly reduction of excretion of fecal anaerobic bacteria and no changes in excretion of beneficial bacteria. The herbs stimulated the production of IL-2, IL-10, TNF, and IFN-g, improving the Ig antibody level in serum, thereby promoting daily weight gain	[123,124]
*Z. officinale*	Ginger	Rhizomes		
*C. longa* Linn.	Turmeric	Rhizomes		

TNF=Tumor necrosis factor, IL=Interleukin, INF=Interferon, *C. longa=Curcuma longa, A. sativum=Allium sativum, R. officinalis=Rosmarinus officinalis, T. vulgaris=Thymus vulgaris, C. verum=Cinnamomum verum, U. dioica=Urtica dioica, I. helenium=Inula helenium, C. majus=Chelidonium majus, T. serpyllum=Thymus serpyllum, C. sativum=Coriandrum sativum, A. hookeri=Allium hookeri, M. stenopetala=Moringa stenopetala, Z. officinale=Zingiber officinale, C. zedoaria=Curcuma zedoaria, A. purpurata=Alpinia purpurata, K. galangal=Kaempferia galangal, G. glabra=Glycyrrhiza glabra, T. vulgare=Tanacetum vulgare, A. vera=Aloe vera, C. aromatic=Curcuma aromatic, T. foenum=Trigonella foenum*

## Anticancer

Recent studies on cancer have bolstered our medical understanding. Cancer occurs not only in humans but also in animals and is frequently described as cellular mutation to DNA within cells because of free radicals in the body affected by exposure to ionizing radiation and other experimental toxins [125]. In avian species, cancer is often related to viruses, namely, avian leukosis virus, Marek’s disease virus, reticuloendotheliosis virus, and lymphoproliferative disease viruses [126].

Chemotherapy is considered the sole treatment for cancer. The genes inside the nucleus of cells will be damaged. Some chemotherapeutic drugs operate by splitting cells, while others aim to inhibit the process of gene replication before cell splitting. In some cases, however, chemotherapy can damage healthy cells and release a protein that supports tumor growth [127].

Herbs can be used in various ways to support cancer treatment by relieving the undesirable effects of chemotherapy, accommodating convalescence after chemo- and radio-therapeutics, promoting the conventional cancer treatment, supporting conventional treatment in some conditions, assisting in cancer prevention, strengthening immune function, and promoting various tracts influenced by cancer [128].

Grape seed proanthocyanidin extract is a polyphenolic antioxidant with pharmacological and nutraceutical benefits, including anticancer properties [129]. Proanthocyanidins can scavenge free radicals 20-fold better than other antioxidants, such as Vitamin C, Vitamin E, and b-carotene, and have potential as cancer growth inhibitors with significantly greater protection against free radicals, free radical-induced lipid peroxidation, and DNA damage [130]. Moreover, seed grape oil has previously shown antioxidant components and effectively worked as a natural antioxidant in broilers [131].

Natural killer (NK) cells known as innate immune cells can eradicate tumor cells [132] by delivering cytotoxic granules containing perforin and granzymes, which lyse target cells [133]. Ashwagandha (*W. somnifera* Dunal) may improve NK cell function in ovarian tumor in laying hen model to diminish occurrence and development of ovarian tumor tissue [134]. The plant has cytolytic properties to fight human tumor cell lines [135,136]. Other herbs that promote NK cells activity are found within the combination of *W. somnifera*, *Glycyrrhiza glabra*, *Z. officinale*, *O. sanctum*, and *Elettaria cardamomum*, which can be fortified in regular tea [137].

A formulation of dandelion root, mustard leaf, and safflower leaf can improve innate chicken immunity, including peripheral blood lymphocyte proliferation, nitric oxide production by macrophages, and free radical scavenging activity, as well as prevent chicken tumor cell development [138]. Other studies showed that safflower (*Carthamus tinctorius*) can augment natural immunity and medicate cancers. Safflower petals contain polysaccharides that augment splenocyte proliferation and stimulate macrophage activation, as well as significantly diminish the viability of chicken tumor cells [139].

Turmeric (*C. longa* Linn.) contains high levels of antioxidants and anti-inflammatory properties [140]. Daily curcumin intake produces a significant and dose-dependent reduction in spontaneous ovarian cancer occurrence and tumor growth, presenting a recommended chemopreventive strategy for ovarian cancer [141]. Various molecular mechanisms revealed that NF-kB and STAT3 signaling pathways were significantly inhibited. However, the nuclear factor erythroid 2/heme oxygenase 1 antioxidant pathway was stimulated by curcumin intake in a dose-dependent manner in ovarian tissue [141]. A compilation of herbs that have anticancer properties for poultry is shown in Table-10.

**Table-10:** List of herbal extract acts as anticancer in poultry.

Plants (species)	Botanical name	Parts used	Mode of action	Reference
*V. vinifera*	Grape	Seeds	Proanthocyanidins are able in scavenging the free radical, indicated as potential for cancer growth inhibition and significantly greater protection against free radicals, free radical-induced lipid peroxidation and DNA damage. Moreover, seed grape oil indicated antioxidant substances and worked effectively as origin of natural antioxidants to broiler	[128, 129, 130]
*W. somnifera* Dunal	Ashwagandha	Roots	Improves the NK cell function in ovarian tumor in the laying hen model that diminished the occurrence and development of ovarian tumor. The plant has cytolytic effect that applicable against human tumor cell lines	[133-135]
*G. glabra*	Liquorice	Roots	Promote NK cells activity that are in the combination with *W. somnifera*, that fortified in a regular tea	[136]
*Z. officinale*	Ginger	Rhizomes		
*O. sanctum*	Tulsi	Leaves		
*E. cardamomum*	Cardamom	Fruits		
*Taraxacum* F.H *Wigg.*	Dandelion	Roots	Affecting chicken innate immunity including peripheral blood lymphocyte proliferation, nitric oxide production by macrophages and free radical scavenging activity; and preventing chicken tumor cell development	[137]
*B. juncea*	Mustard	Leaves		
*C. tinctorius*	Safflower	Leaves		
*C. tinctorius*	Safflower leaf	Leaves	Augment natural immunity and medicate cancers. The safflower petals contain polysaccharides that augmented splenocyte proliferation and stimulated macrophages activation, and significantly diminished the viability of chicken tumor cell	[138]
*C. longa* Linn.	Turmeric	Rhizomes	Contain high of antioxidant and anti-inflammatory properties The plant inhibits NF-kB and STAT3 signaling pathways, however, that the nuclear factor erythroid 2/hemeoxygenase 1 antioxidant pathway was stimulated by curcumin intake in a dose-dependent manner in ovarian tissues	[139] [140,141]

NK=Natural killer, *W. somnifera=Withania somnifera, O. sanctum=Ocimum sanctum, C. longa=Curcuma longa, E. cardamom=Elettaria cardamom, C. tinctorius=Carthamus tinctorius, V. vinifera=Vitis vinifera, G. glabra=Glycyrrhiza glabra, Z. officinale=Zingiber officinale, B. juncea=Brassica juncea*

## Conclusion

This review demonstrated that certain plants are potential medicines for avian species. Various plants present numerous advantages, including minimal side effects potentially harmless for birds, humans, and the environment, as well as limiting drug resistance and diminishing residue in chicken meat and eggs. Further studies on other medicinal plants applied to chickens are needed to explore the potential of other plants on avian health. In the future, natural products with low side effects to fight poultry disease may be possible; moreover, some possible limitations in this review can exist. More methodological work is needed to offer more medicinal plants as alternatives to synthetic drugs that are effective and safe for poultry, humans, and the environment.

## Authors’ Contributions

TH, MRAAS, and RLB: Conceptualized and designed the review. TH and MRAAS: Collected the literatures. TH: Analyzed the data. TH and SJ: Drafted the manuscript. RLB, BPP, and SJ: Edited and finalized the manuscript. All authors read and approved the manuscript.
